# Supplementary material for: Identification and functional analysis of non-coding regulatory small RNA FenSr3 in Bacillus amyloliquefaciens LPB-18
Source: PeerJ. 2023 May 15;11:e15236. doi: 10.7717/peerj.15236 (PMC10194069; doi:10.7717/peerj.15236)
Supplement: Supplemental Information 4 [file peerj-11-15236-s004.zip › KO/CK-vs-T1_map/map04960.html]

KEGG PATHWAY: Aldosterone-regulated sodium reabsorption - Reference pathway


|  |  |
| --- | --- |
| **Aldosterone-regulated sodium reabsorption - Reference pathway** |  |

[
Pathway menu
| Organism menu
| Pathway entry
| Show description
| User data mapping
]

Sodium transport across the tight epithelia of Na+ reabsorbing tissues such as the distal part of the kidney nephron and colon is the major factor determining total-body Na+ levels, and thus, long-term blood pressure. Aldosterone plays a major role in sodium and potassium metabolism by binding to epithelial mineralocorticoid receptors (MR) in the renal collecting duct cells localized in the distal nephron, promoting sodium resorption and potassium excretion. Aldosterone enters a target cell and binds MR, which translocates into the nucleus and regulates gene transcription. Activation of MR leads to increased expression of Sgk-1, which phosphorylates Nedd4-2, an ubiquitin-ligase which targets ENAC to proteosomal degradation. Phosphorylated Nedd4-2 dissociates from ENAC, increasing its apical membrane abundance. Activation of MR also leads to increased expression of Na+/K+-ATPase, thus causing a net increase in sodium uptake from the renal filtrate. The specificity of MR for aldosterone is provided by 11beta-HSD2 by the rapid conversion of cortisol to cortisone in renal cortical collecting duct cells. Recently, besides genomic effects mediated by activated MR, rapid aldosterone actions that are independent of translation and transcription have been documented.

|  |  |  |
| --- | --- | --- |
| Reference pathway | 184% 150% 122% 100% 82% 67% 55% | 图片下载 |
